# Are polar bear habitat resource selection functions developed from 1985–1995 data still useful?

**DOI:** 10.1002/ece3.5401

**Published:** 2019-07-04

**Authors:** George M. Durner, David C. Douglas, Todd C. Atwood

**Affiliations:** ^1^ Alaska Science Center U.S. Geological Survey Anchorage Alaska USA; ^2^ Alaska Science Center U.S. Geological Survey Juneau Alaska USA

**Keywords:** Arctic, climate change, habitat, polar bear, resource selection functions, sea ice, *Ursus maritimus*

## Abstract

1. Greenhouse‐gas‐induced warming in the Arctic has caused declines in sea ice extent and changed its composition, raising concerns by all circumpolar nations for polar bear conservation.
2. Negative impacts have been observed in three well‐studied polar bear subpopulations. Most subpopulations, however, receive little or no direct monitoring, hence, resource selection functions (RSF) may provide a useful proxy of polar bear distributions. However, the efficacy of RSFs constructed from past data, that is, reference RSFs, may be degraded under contemporary conditions, especially in a rapidly changing environment.
3. We assessed published Arctic‐wide reference RSFs using tracking data from adult female polar bears captured in the Beaufort Sea. We compared telemetry‐derived seasonal distributions of polar bears to RSF‐defined optimal sea ice habitat during the period of RSF model development, 1985–1995, and two subsequent periods with diminished sea ice: 1996–2006 and 2007–2016. From these comparisons, we assessed the applicability of the reference RSFs for contemporary polar bear conservation.
4. In the two decades following the 1985–1995 reference period, use and availability of optimal habitat by polar bears declined during the ice melt, ice minimum, and ice growth seasons. During the ice maximum season (i.e., winter), polar bears used the best habitat available, which changed relatively little across the three decades of study. During the ice melt, ice minimum, and ice growth seasons, optimal habitat in areas used by polar bears decreased and was displaced north and east of the Alaska Beaufort Sea coast. As optimal habitat diminished in these seasons, polar bears expanded their range and occupied greater areas of suboptimal habitat.
5. Synthesis and applications: Sea ice declines due to climate change continue to challenge polar bears and their conservation. The distribution of Southern Beaufort Sea polar bears remained similar during the ice maximum season, so the reference RSFs developed from data collected >20 years ago continue to accurately model their winter distribution. In contrast, reference RSFs for the ice transitional and minimum seasons showed diminished predictive efficacy but were useful in revealing that contemporary polar bears have been increasingly forced to use suboptimal habitats during those seasons.

## 1 INTRODUCTION

**Figure 1:**
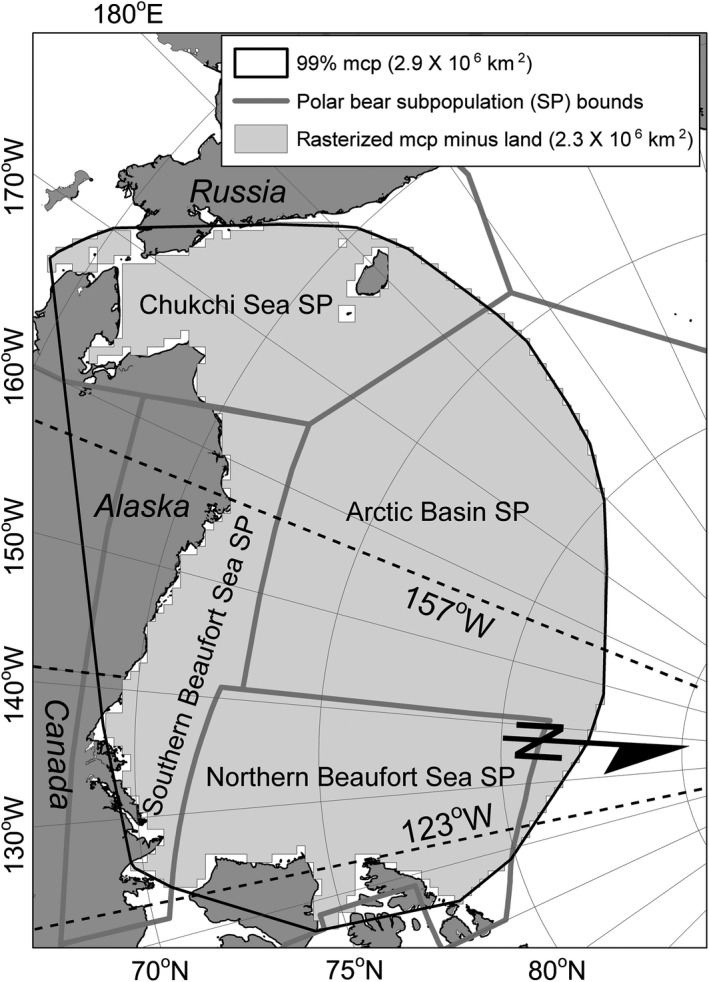
Study area defined by a rasterized 99% minimum convex polygon (mcp) of satellite telemetry locations from adult female polar bears radio‐tagged between 123° and 157° W longitude, 1985–2016. Also shown is the overlap between the study area and polar bear subpopulations (SP) as defined by the International Union for Conservation of Nature (Durner et al., 2018). 5° of latitude = 556 km

Since at least the mid‐1990s, polar bears (*Ursus maritimus*) have contended with an environment that is rapidly changing due largely to greenhouse‐gas‐induced climate warming (Atwood et al., 2016). Polar bears evolved as specialized predators of sea ice‐associated seals (e.g., ringed seals [*Pusa hispida*] and bearded seals [*Erignathus barbatus*]) and because of this occur only where the surface of Northern Hemisphere marine waters is >50% sea ice for a substantial portion of the year (Stern & Laidre, 2016). Since 1980, global atmospheric CO_2_ has risen from ~340 ppm to >410 ppm (https://www.esrl.noaa.gov/gmd/ccgg/trends/global.html; accessed 13 April 2019). Concurrently, average global air temperature rose > 1°C above preindustrial levels for the first time in 2015 (Hawkins et al., 2017). Arctic sea ice declines have been directly related to greenhouse gas emissions (Notz & Stroeve, 2016), and since 1978 ice extent declines have ranged from 3.0% decade^−1^ in March to 12.8% decade^‐1^ in September, (http://nsidc.org/arcticseaicenews/; accessed 13 April 2019). Across the Arctic, the presence of seasonal sea ice decreased 5 days decade^−1^ between 1979 and 2013 (Parkinson, 2014). Declines in the spatial and temporal distribution of sea ice have been accompanied by reductions in ice age and thickness. During 1985 to 2015, first year ice in March increased from 50% to 70% of total pack ice extent while ice >4 years old decreased from 20% to 3% (Tschudi, Stroeve, & Stewart, 2016). The twelve lowest summertime sea ice extents ever recorded occurred during 2007 to 2018 (http://nsidc.org/arcticseaicenews/2018/09/; accessed 13 April 2019). Since the early 2000s, synergistic interactions between decreasing ice thickness, increased mobility and fracturing, and reduced surface albedo have rendered the ice pack more vulnerable to melting (Kashiwase, Ohshima, Nihashi, & Eicken, 2017). These changes have negatively impacted polar bear sea ice habitat (Stern & Laidre, 2016). Since 1979 within the region including the Southern Beaufort Sea polar bear subpopulation (SB; Figure 1), the ice retreat date has occurred 9.0 days decade^−1^ earlier and the ice advance date 8.8 days decade^−1^ later (Stern & Laidre, 2016). A significant change in ice retreat (4.0 days decade^−1^ earlier) and advance (5.3 days decade^−1^ later) has also occurred in the adjacent region that includes the Chukchi Sea polar bear subpopulation (Figure 1; Stern & Laidre, 2016).

Because polar bears live in an environment that is rapidly changing, they are the focus of conservation efforts by all nations whose jurisdictions overlap their range (Durner, Laidre, & York, 2018). Nineteen subpopulations of polar bears are recognized (Durner et al., 2018), and all have experienced loss of sea ice habitat since 1979 (Stern & Laidre, 2016). Of those subpopulations with sufficient monitoring, three have undergone population declines (Bromaghin et al., 2015; Lunn et al., 2016; Obbard et al., 2018), two have undergone range contractions (Laidre et al., 2018) or shifts in habitat use (Laidre et al., 2015), and five have shown no apparent negative impacts (Peacock, Taylor, Laake, & Stirling, 2013; Regehr et al., 2018, Stapleton, Peacock, & Garshelis, 2016, Stirling, McDonald, Richardson, Regehr, & Amstrup, 2011, SWG, 2016). Responses of the remaining nine polar bear subpopulations are unknown (Durner et al., 2018); hence, information to assist conservation decisions is currently unavailable in much of the range of polar bears. As degradation of the sea ice habitat used by polar bears continues through the 21st century (Durner et al., 2009), understanding how polar bears distribute themselves relative to sea ice composition may serve as a useful proxy for assessing their conservation status (Vongraven et al., 2012).

Resource selection functions (RSF) model the relationship between animal locations and environmental covariates with the goal of estimating the relative probability of a resource being used (Manly, McDonald, Thomas, McDonald, & Erickson, 2002). As an index of habitat suitability, RSFs can quantify the likely distribution of a population across its range (Boyce et al., 2017) and may be used to estimate changes in habitat quality resulting from environmental change (Durner et al., 2009). RSFs are often structured as discrete choice models, where the set of resources available to an individual is allowed to vary between individuals and across choice sets (Arthur, Manly, McDonald, & Garner, 1996; McDonald, Manly, Nielson, & Diller, 2006). As such, discrete choice RSFs are relatively robust to variation in habitat composition (Arthur et al., 1996). Nevertheless, the efficacy of RSFs applied at a future time should be verified, especially if environmental conditions have markedly changed (Garshelis, 2000). This is true for polar bears whose sea ice habitats have changed (Stern & Laidre, 2016) and, in the Beaufort Sea, may now use larger areas because movement rates have increased to compensate for faster sea ice drift in recent years (Auger‐Méthé, Lewis, & Derocher, 2015; Durner et al., 2017). Verification is especially important when the target species is of conservation concern and efforts to monitor populations are either deficient, intermittent, or nonexistent; all of which are factors in the international attempts to monitor the 19 subpopulations of polar bears (Vongraven et al., 2012).

In 2007, to inform a decision by the U.S. Secretary of the Interior on whether to list polar bears as a threatened species throughout their range, the U.S. Geological Survey (USGS) launched a suite of comprehensive studies to assess the long‐term impact of a changing Arctic on polar bears. One component of that work involved an international collaboration to assess observed and predicted changes in polar bear sea ice habitat throughout the Arctic Ocean and adjoining peripheral seas (Durner et al., 2009). Using satellite telemetry locations of adult female polar bears between 1985–1995, Durner et al. (2009) developed seasonal RSFs that showed, Arctic‐wide, optimal habitat declined within the ranges of most polar bear subpopulations in the following decade (1996–2006) and was projected to decline in all subpopulations by the end of the 21st century. Further, Durner et al. (2009) suggested that the RSF, when applied Arctic‐wide and pooled over seasons, maintained efficacy during 1996–2006, as 82.3% of polar bear locations occurred in the upper 20% of RSF‐valued sea ice habitat. This suggested that sea ice characteristics selected by polar bears, as measured from passive microwave imagery of sea ice concentration and extent (25 × 25 km pixels; National Snow and Ice Data Center; http://nsidc.org/), remained fairly consistent across those two decades despite declining ice trends. The assessment by Durner et al. (2009), however, did not consider decadal changes in RSF performance within subregions of the Arctic nor during specific seasons.

Philopatry is evident in polar bear subpopulations (Amstrup, McDonald, & Durner, 2004; Bethke, Taylor, Amstrup, & Messier, 1996; Mauritzen et al., 2002), and sea ice changes have regional specificity (Stern & Laidre, 2016). Because governmental polar bear conservation efforts are typically focused on the regions within their jurisdictions, a regional and seasonally focused approach to inform those efforts is needed. Additionally, steep rates of Arctic summer sea ice loss have continued (Stroeve & Notz, 2018) since the period that Durner et al. (2009) used to assess their RSF, raising questions about the model's efficacy for understanding the spatial ecology of polar bears in the contemporary Arctic and for informing present‐day conservation actions. In this paper, we present an assessment of the RSFs developed by Durner et al. (2009) when applied to later periods in the Beaufort and Chukchi seas. Although sea ice conditions have been continuously changing since 1979 (Stroeve & Notz, 2018), we set the 1985–1995 Arctic‐wide RSFs developed by Durner et al. (2009) as a baseline for comparison (hereafter referred to as reference RSFs). We apply the reference RSFs to sea ice conditions during the 1985–1995 reference period and to two subsequent decadal periods, 1996–2006 and 2007–2016. We compare the distribution of radio‐tagged adult female polar bears to the distribution of RSF‐valued habitat as modeled by the reference RSFs in each of four seasons and three decadal periods. The analyses address three objectives: (a) assess the decadal and seasonal associations of polar bear distributions with RSF‐valued habitat as defined by reference RSFs; (b) determine the amount and distribution of optimal habitat within regions used by polar bears across decades and seasons; and (c) determine whether RSF models developed under different sea ice regimes have value to researchers and managers in measuring progress toward meeting polar bear conservation goals.

## 2 METHODS

### 2.1 Polar bear location data

From 1985 to 2016, we captured adult female polar bears in the southern Beaufort Sea and equipped them with either collar, ear tag, or glue‐on platform transmitter terminals (PTT) that provided Doppler‐derived or GPS locations. We restricted our analysis to data from adult female polar bears because adult male and subadult polar bears cannot wear radio collars, and the vast majority of tracking data has been of adult females. We retained data from a PTT if it was deployed on a bear captured between 123° and 157° west longitude (Figure 1). We performed an initial filter to remove implausible Doppler locations using the Douglas Argos‐filter (DAF) algorithm (Douglas et al., 2012). DAF retained all standard‐quality locations (Argos location classes 3, 2, and 1), and auxiliary location classes (0, A, and B) when corroborated by a consecutive location within a 10 km radius, or when movement rates were <10 km/hr and the internal angles (α, in degrees) formed by preceding and subsequent vectors (of lengths d1 and d2 km) were not suspiciously acute (*α* > −25 + *β* × ln[minimum (d1,d2)], where *β* = 15). We assigned *β* = 15 because it performed well for our specific tracking data across seasons and regions. We excluded locations of bears that were on land or in maternal dens, and from PTTs that had become detached as evidenced by invariant activity sensor data, temperature sensor data emulating ambient conditions, or location data either persistently stationary or persistently following the prevailing ice drift.

### 2.2 Reference RSFs and environmental data

For the reference RSFs, we used the four seasonal RSFs (winter, spring, summer, and autumn), the four environmental covariates (sea ice concentration [SIC], distance to the 15% SIC interface, ocean depth, and distance to land), and the original coefficients (Table 1) that were published by Durner et al. (2009). Durner et al. (2009) found that the four covariates were highly predictive of polar bear distribution, with bears selecting for sea ice of medium to high concentration, over shallow seas, near coasts, and in close proximity to low SIC. Identical to Durner et al. (2009), we obtained monthly estimates of SIC from the National Snow and Ice Data Center (NSIDC; 25 × 25 km pixel size, polar stereographic projection; Cavalieri, Parkinson, Gloersen, & Zwally, 1996; accessed 15 August 2017) and converted them to Arc/Info grids (ver. 9.3; ESRI). Although polar bears may respond to many sea ice metrics (e.g., see Ferguson, Taylor, & Messier, 2000), NSIDC estimates of sea ice concentration derived from 25 km resolution passive microwave sensors were the only data consistently available throughout the Northern Hemisphere during the years examined by Durner et al. (2009). Likewise, ocean depth and distance to land are both invariant across years and therefore provided a consistent source of environmental data during the entire study period.

**Table 1:** Coefficients and standard errors (in parentheses) of covariates in four seasonal resource selection functions for polar bears in the polar basin, 1985–1995, reported Durner et al. (2009) and used in this report

Season	Ice concentration	Ice concentration squared	Ocean depth	Distance to land	Distance to 15% ice concentration threshold
Winter	0.08602 (0.01856)	−0.00046 (0.00012)	−0.00037 (0.00006)	−0.00474 (0.00047)	
Spring	0.06551 (0.00409)	−0.00040 (0.00004)	−0.00020 (0.00005)		−0.00261 (0.00050)
Summer	0.04676 (0.00582)	−0.00037 (0.00007)	−0.00017 (0.00005)		−0.00436 (0.00083)
Autumn	0.08130 (0.00635)	−0.00068 (0.00006)	−0.00025 (0.00005)		−0.00604 (0.00054)

Note

See Durner et al. (2009) for details.

We obtained ocean depth from the International Bathymetric Chart of the Arctic Ocean (IBCAO) Version 3.0 (pixel size: 0.5 × 0.5 km; Jakobsson et al., 2012). A GIS line coverage of the coast was derived by contouring the depth grid at cell values equal to zero. We created a distance to land grid by calculating the distance from each SIC pixel to its nearest point on the coastline coverage. All grids were converted to Lambert equal area projection with 25 × 25 km cell size.

### 2.3 Study area

We defined our study area as the 99% minimum convex polygon (MCP) encompassing all polar bear locations, 1985–2016. One location per day per bear was selected for the MCP analysis based on the best location quality (GPS, then Argos quality classes 3, 2, 1, 0, A, and B); ties were decided randomly. A single MCP was derived from the daily polar bear locations using the mcp function in the R package adehabitatHR (Calenge, 2017). The MCP polygon was converted to a grid with the same map projection and pixel size as the grids of environmental data. Land cells within the MCP were omitted from the study area. The resulting study area included the extent of four of the 19 recognized subpopulations of polar bears, that is, the SB, Arctic Basin, Chukchi Sea, and Northern Beaufort Sea (Durner et al., 2018).

### 2.4 Period assignments

Identical to Durner et al. (2009), we used 1985–1995 as the reference period from which to make comparisons with subsequent periods. Durner et al.'s (2009) decision to separate 1985–1995 from 1996 to 2006 was based on (a) reduced sampling effort in 1995 and 1996, (b) reduced sea ice extent during 1996–2006 relative to 1985–1995, and (c) better representation of tracking data in all polar bear subpopulations in the Arctic basin in 1985–1995 than in 1996–2006. We used the same reference period to establish a minimum RSF‐value threshold for optimal polar bear habitat in each season within the MCP study area. To assess the reference RSFs during conditions of sea ice decline, we divided the remaining years into two decadal periods: 1996–2006, years that had substantially lower sea ice extent relative to 1985–1995 (Ogi & Wallace, 2007), and 2007–2016, which included 10 of the 12 lowest annual minimum Arctic sea ice extents in the satellite record (NSIDC, http://nsidc.org/arcticseaicenews/2018/09/arctic-sea-ice-extent-arrives-at-its-minimum/, accessed 26 February 2019).

### 2.5 Season assignments

We assigned monthly SIC grids to one of four temporally dynamic seasons (i.e., melt, minimum, growth, and maximum) using the methods from Durner et al. (2009). Season length was allowed to vary across years to accommodate the Arctic's changing seasonality. During any given year, a month was assigned to the maximum season when sea ice extent within our study area was greater than the annual maximum extent minus 15% of the respective year's maximum–minimum amplitude. Conversely, a month was assigned to the minimum season if its sea ice extent was less than the annual minimum extent plus 15% of the respective year's maximum–minimum amplitude. Months between the maximum season and minimum season were assigned to either the ice melt season or the ice growth season depending on the time of year (Durner et al., 2009).

### 2.6 Calculating RSF grids and equal area zones

Monthly RSF grids were derived using the appropriate seasonal model (Table 1) with the respective monthly grids of SIC, distance to 15% ice concentration interface, and the two invariant grids of distance to land and ocean depth. RSF cells were set to missing data when SIC was <15% concentration (i.e., ice‐free). Excluding SIC < 15% was based on the diminished reliability of the SIC estimates under conditions when the passive microwave signatures are dominated by open water (Meier, Fetterer, Stewart, & Helfrich, 2015). Defining cells with SIC < 15% as ice‐free likely excluded instances where small amounts of ice were present and possibly occupied by some polar bear locations. Each derived monthly RSF grid was binned into 20 equal area zones based on nonmissing RSF values. Summary metrics about each zone were calculated, including pixel count, total area, and the minimum RSF value.

### 2.7 Assigning RSF zones to polar bear locations

We calculated the percentage of polar bear locations within each equal area RSF zone using only higher‐accuracy locations (GPS and Argos classes 3, 2, and 1) that were no less than 72 hr apart (to reduce autocorrelation and standardize interannual sampling intensity). Locations that occurred outside of RSF zones were excluded. For each decadal period, the seasonal mean percentage of bear locations within each equal area interval and 95% confidence intervals were calculated by bootstrapping. For each of 25 bootstrap iterations, we set the sample size to the number of bears within the respective period and season.

### 2.8 Assessing polar bear responses to changes in optimal habitat

Durner et al. (2009) chose the upper 20% of RSF equal area zones during 1985–1995 as the reference definition for “optimal” polar bear habitat. That upper RSF‐valued zone included 71.6% of all polar bear telemetry locations during 1985–1995 across all seasons Arctic‐wide (10.8 × 10^6^ km^2^). We emulated that method but calculated it for our study area (2.3 × 10^6^ km^2^). We averaged the lower RSF threshold‐value for the 17th equal area bin (i.e., the 80th percentile) across all months for each season and every year during 1985–1995. We then calculated a single average for each season to establish the lower threshold of optimal polar bear habitat for the reference period and applied those thresholds to the respective seasonal RSF grids of subsequent periods.

To assess optimal habitat within an area overlapping the spatial distribution of polar bears, we developed kernel utilization distributions (UD) of polar bear locations for each decadal period and season. We defined an overall area of polar bear occupancy as the region that encompassed 95% of the UD, and a “core” area that included 50% of the UD. We used the same higher‐quality, temporally restricted subset of polar bear locations described above to generate period‐season UDs with function kernelUD in the R package adehabitatHR (ver. 0.4.15; Calenge, 2017). Total area for each period‐season UD that was within the study area was calculated. Period‐season UD grids were then matched with their respective monthly RSF optimal habitat grids to extract the area of optimal habitat within the UDs for each month. The percent of optimal habitat within UDs for each month was derived by dividing UD optimal habitat area by the total UD area and multiplying by 100. Season‐specific percentages of optimal habitat were compared between periods with boxplots and ANOVAs followed by post hoc Tukey HSD tests. We used chi‐square tests to examine proportional changes in polar bear occupancy of high RSF‐valued habitat defined by the reference RSFs when applied to 1996–2006 and 2007–2016. Changes in the proportion of polar bear locations were evaluated for two categories of high‐quality habitat: (a) equal area intervals ≥10, that is the upper 50% of RSF‐valued habitat; and (b) equal area intervals ≥ 17, that is, the top 20% of RSF‐valued habitat. For the chi‐square tests we reduced the number of polar bear locations to one per month per individual to constrain pseudoreplication. This was accomplished by selecting the best quality location(s) for each bear each month and, in the case of ties, randomly selecting only one of those records. Significance for all tests was set to *α* < 0.05.

We used centroids of optimal habitat within 95% kernel UDs to examine whether the distribution of optimal habitat has changed spatially across periods. Centroids for each period‐season were derived by averaging the coordinates of all pixels in each monthly RSF that occurred within the respective 95% kernel UD. We then transformed those coordinate averages to longitude and latitude and used the distHaversine and bearing functions in R package geosphere (ver. 1.5‐7; Hijmans, 2017) to, respectively, calculate the great‐circle‐distance and initial bearing from each centroid to the centroid in the next period (e.g., melt 1985–1995 to melt 1996–2006, and melt 1996–2006 to melt 2007–2016).

## 3 RESULTS

After imposing filters, 56,977 locations from 301 bears were used to define a 99% MCP study area which encompassed 2,298,125 km^2^ after rasterizing and excluding cells over land (Figure 1). After retaining only higher‐quality locations and excluding those that were <72 hr apart, there were (records/individuals for kernel UDs) 6,211/93 for 1985–1995, 7,641/97 for 1996–2006, and 7,766/127 for 2007–2016. Sum of period totals for individual bears (317) was greater than 301 because some bears occurred in two periods. A summary of period and season sample sizes is provided in Appendix 1.

Beginning in May 1985 and ending in December 2016, a total of 380 NSIDC grids of monthly SIC were analyzed. Of these, 230 were classified as ice maximum, 63 as ice melt, 42 as ice minimum, and 45 as ice growth season.

### 3.1 Seasonal polar bear locations relative to RSF‐valued zones

#### 3.1.1 Ice melt

**Figure 2:**
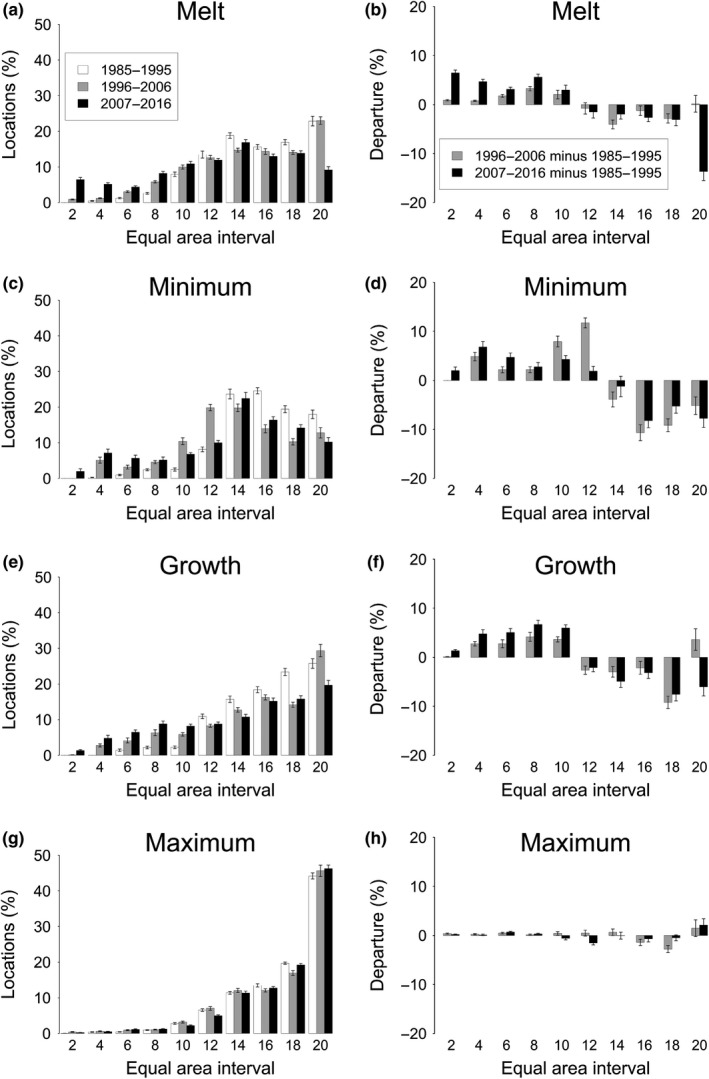
(Left side) Percentages of polar bear telemetry locations (% ± 95% CI) that occurred within 10 equal area RSF zones of increasing RSF value, by period and season: ice melt (a), minimum (c), growth (e), and maximum (g). (Right side) Departures (% ± 95% CI) of the 1996–2006 and 2007–2016 proportions from 1985 to 1995 reference RSF, for the ice melt (b), minimum (d), growth (f), and maximum (h) seasons. Sequential 5% equal area zones were merged into 10% zones to aid visualization

The mean length of the ice melt season from 1985 to 2016 was 2.0 months (range: minimum = 0, maximum = 3). A smaller proportion of polar bear locations in 1996–2006 and 2007–2016, relative to 1985–1995, often occurred in the highest RSF equal area zones during the ice melt season (Figure 2a,b). During 1985–1995, the proportion of bear locations in the upper 50% and 20% of the RSF‐valued habitat was 0.87 and 0.36, respectively (Table 2). Relative to 1985–1995, the proportion of locations in the upper 50% of RSF‐valued habitat in 1996–2006 was significantly smaller (proportion = 0.74, Table 2) but not significantly different in the upper 20% of RSF‐valued habitat (proportion = 0.32). Relative to 1985–1995, the proportion of locations in both the upper 50% and upper 20% of RSF‐valued habitat in 2007–2016 were significantly smaller (upper 50%: proportion = 0.59; upper 20%: proportion = 0.21; Table 2).

**Table 2:** Proportion of polar bear locations in the Beaufort and Chukchi seas occurring in the upper 20% and upper 50% of RSF‐valued habitat for 1996–2006 and 2007–2016, compared with a chi‐square test of proportions to the proportion of locations during the reference period (1985–1995)

Season	1985–1995	1996–2006			2007–2016		
	Proportion	Proportion	*χ* ^2^	*p*	Proportion	*χ* ^2^	*p*
Upper 20% of RSF‐valued habitat							
Melt	0.36	0.32	0.965	0.326	0.21	10.226	0.001
Minimum	0.41	0.34	1.373	0.241	0.21	10.114	0.001
Growth	0.51	0.44	1.994	0.158	0.39	4.143	0.042
Maximum	0.65	0.63	1.064	0.302	0.68	1.221	0.269
Upper 50% of RSF‐valued habitat							
Melt	0.87	0.74	12.767	<0.001	0.59	37.827	<0.001
Minimum	0.92	0.82	6.892	0.009	0.77	11.843	<0.001
Growth	0.91	0.78	10.734	0.001	0.77	10.927	<0.001
Maximum	0.95	0.93	3.622	0.057	0.93	2.089	0.148

#### 3.1.2 Ice minimum

The mean length of the ice minimum season from 1985 to 2016 was 1.3 months (range: minimum = 1, maximum = 2). The ability of the RSF to predict the distribution of polar bears during the ice minimum season after the reference period of 1985–1995 was often diminished (Figure 2c,d). During 1985–1995, the proportion of bear locations in the upper 50% and 20% of the RSF‐valued habitat was 0.92 and 0.41, respectively (Table 2). Relative to 1985–1995, the proportion of locations in the upper 50% of RSF‐valued habitat in 1996–2006 was significantly smaller (proportion = 0.82) but not significantly smaller in the upper 20% of RSF‐valued habitat (proportion = 0.34; Table 2). Relative to 1985–1995, the proportion of locations in both the upper 50% and upper 20% of RSF‐valued habitat in 2007–2016 were significantly smaller (upper 50%: proportion = 0.77; upper 20%: proportion = 0.21; Table 2).

#### 3.1.3 Ice growth

The mean length of the ice growth season from 1985 to 2016 was 1.4 months (range: minimum = 1, maximum = 2). Like the ice melt and ice minimum seasons, there was often diminished ability of the RSF to predict the distribution of polar bears during the ice growth season after the reference decade of 1985–1995 (Figure 2e,f). During 1985–1995, the proportion of bear locations in the upper 50% and 20% of the RSF‐valued habitat was 0.91 and 0.51, respectively. Relative to 1985–1995, the proportion of locations in the upper 50% of RSF‐valued habitat in 1996–2006 was significantly smaller (proportion = 0.78) but not significantly smaller in the upper 20% of RSF‐valued habitat (proportion = 0.44; Table 2). Relative to 1985–1995, the proportion of locations in both the upper 50% and upper 20% of RSF‐valued habitat in 2007–2016 were significantly smaller (upper 50%: proportion = 0.77; upper 20%: proportion = 0.39; Table 2).

#### 3.1.4 Ice maximum

The mean length of the ice maximum season from 1985 to 2016 was 7.3 months (range: minimum = 6, maximum = 9). The ice maximum season had the best RSF performance and the most consistent performance in decades after the reference period. During ice maximum seasons, the proportion of polar bear locations occurring within equal area bins was similar across periods and CIs often overlapped (Figure 2g,h). During 1985–1995, the proportion of bear locations in the upper 50% and 20% of the RSF‐valued habitat was 0.95 and 0.65, respectively. Relative to 1985–1995, the proportion of locations in the upper 50% of RSF‐valued habitat in 1996–2006 was similar (proportion = 0.93), as was the upper 20% of RSF‐valued habitat (proportion = 0.63; Table 2). Relative to 1985–1995, the proportion of locations in the upper 50% of RSF‐valued habitat in 2007–2016 was similar (proportion = 0.93), as was the upper 20% of RSF‐valued habitat (proportion = 0.68; Table 2).

### 3.2 Assessing the relative distributions of polar bears and optimal habitat

**Figure 3:**
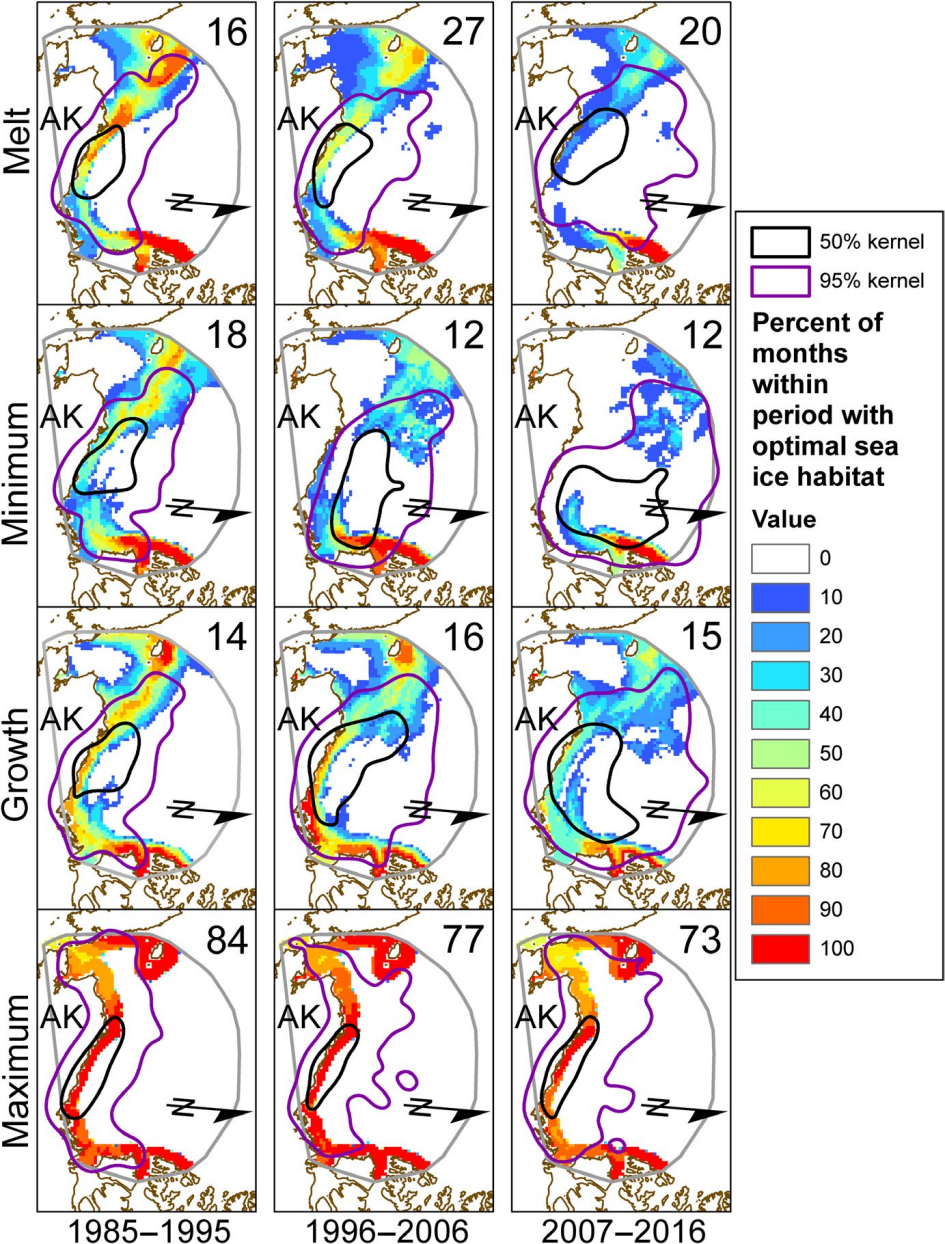
Spatial distribution and month frequency of optimal sea ice habitat presence, in the Beaufort and Chukchi seas, as modeled by reference (1985–1995) polar bear resource selections functions (Durner et al., 2009) for three periods and all months within each season, overlaid with 50% (black) and 95% (purple) kernel utilization distribution polygons of polar bears derived from satellite telemetry locations during each period and season. Number of months is provided in the upper right corner of each panel. Map extent and orientation as in Figure 1, north arrow provided for reference, AK = Alaska

From 1985–1995 to 2007–2016, an increase in space use by polar bears was evident across the study area, as was a reduction in the proportion of the study area that was comprised of optimal habitat. From 1985–1995 to 2007–2016, area of seasonal polar bear 50% kernel UDs increased across decades by 48%–118% in all seasons except the ice maximum (Figure 3, Table 3). Spatial area of the 95% UDs increased in all seasons by as much as 16%–88% (Figure 3, Table 4). In contrast, the proportion of the UD area that was comprised by optimal habitat generally showed a declining trend. From 1985–1995 to 2007–2016, the average percent of monthly optimal habitat during ice melt within 50% UDs declined (*F*_2,60_ = 9.06, *p* < 0.001; Table 3), as optimal habitat in 2007–2016 (3.9%) was less than in 1985–1995 (22.1%) and in 1996–2006 (16.8%; Table 3). During the ice minimum season the percent of optimal habitat within 50% kernel UDs was similar across periods (*F*_2,39_ = 2.09, *p* > 0.05; 1985–1995:12.9%; 1996–2006:10.8%; 2007–2016:8.0%; Table 3). Like the ice melt season, optimal habitat during ice growth within 50% UDs decreased from 1985–1995 to 2007–2016 (*F*_2,42_ = 7.65, *p* < 0.01), as there was less optimal habitat in 2007–2016 (10.1%) than in 1985–1995 (19.4%) and in 1996–2006 (17.3%; Table 3). During the ice maximum season the percent of optimal habitat in 50% UDs increased from 1985–1995 to 2007–2016 (*F*_2,231_ = 8.94, *p* < 0.001). During the maximum ice season, the percent of optimal habitat within the 50% UD was greater in 1996–2006 (50.1%) than in 1985–1995 (45.9%) and greater in 2007–2016 (48.6%) than in 1985–1995 (Table 3).

**Table 3:** Seasonal 50% kernel utilization distribution areas (km^2^) and mean monthly amount (%, *SD*, *n*) of each UD comprised of optimal polar bear sea ice habitat in four seasons and three decadal periods in the Beaufort and Chukchi seas, 1985–2016

Melt				Minimum				Growth				Maximum			
km^2^	%	*SD*	*n*	km^2^	%	*SD*	*n*	km^2^	%	*SD*	*n*	km^2^	%	*SD*	*n*
Period: 1985–1995															
165,625	22.1^A ^	13.0	16	232,500	12.9^A^	9.0	18	211,250	19.4^A^	8.0	14	165,000	45.9^A ^	4.2	84
Period: 1996–2006															
168,125	16.8^A ^	16.4	27	359,375	10.8^A^	4.2	12	375,625	17.3^A^	7.2	16	113,750	50.1^B ^	5.7	77
Period: 2007–2016															
245,625	3.9^B ^	8.9	20	480,625	8.0^A^	2.9	12	461,250	10.1^B^	4.8	15	137,500	48.6^B ^	8.8	73

Note

Significantly different period means (within seasons) are denoted by different superscripted letters (Tukey HSD, *p* < 0.05).

**Table 4:** Seasonal 95% kernel utilization distribution areas (km^2^) and mean monthly amount (%, *SD*, *n*) of each UD comprised of optimal polar bear sea ice habitat in four seasons and three decadal periods in the Beaufort and Chukchi seas, 1985–2016

Melt				Minimum				Growth				Maximum			
km^2^	%	*SD*	*n*	km^2^	%	*SD*	*n*	km^2^	%	*SD*	*n*	km^2^	%	*SD*	*n*
Period: 1985–1995															
872,500	25.5^A ^	9.1	16	976,250	22.5^A ^	6.1	18	855,625	27.1^A ^	4.2	14	888,125	35.1^A ^	8.1	84
Period: 1996–2006															
853,125	11.9^B ^	8.8	27	1,155,000	13.7^B ^	3.9	12	1,279,375	18.9^B ^	2.7	16	853,750	27.8^B ^	6.4	77
Period: 2007–2016															
1,255,625	5.2^C ^	4.7	20	1,591,875	8.0^C ^	3.8	12	1,604,375	13.6^C ^	3.8	15	1,028,750	25.0^B ^	8.4	73

Note

**Figure 4:**
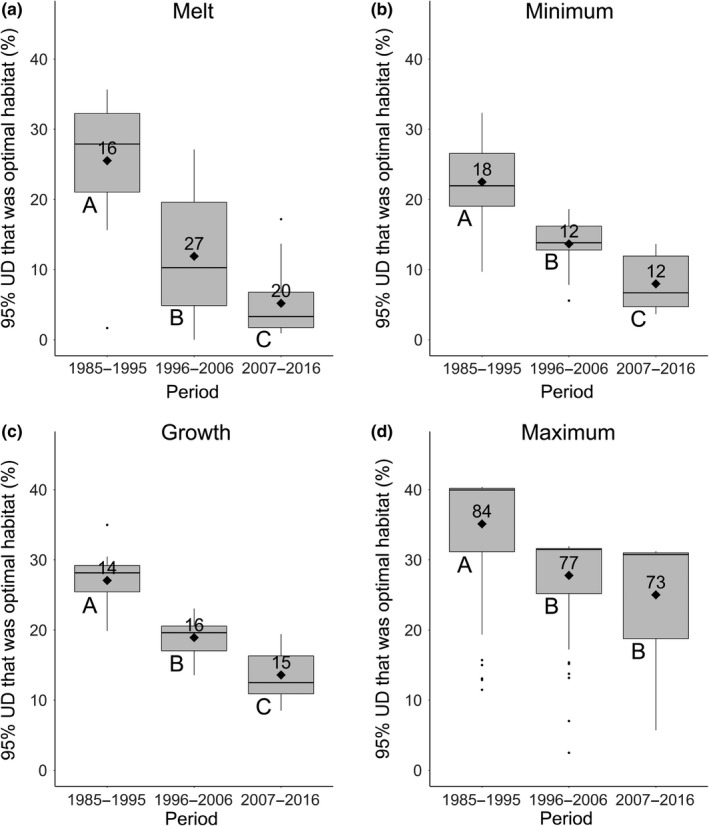
Boxplots (median, quartiles, 5% and 95% whiskers, and outliers) showing the annual proportion of polar bear 95% kernel utilization distributions (UDs) comprised of optimal sea ice habitat in the Beaufort and Chukchi seas within decadal periods during each of four seasons: ice melt (a), ice minimum (b), ice growth (c), and ice maximum (d). Significant differences between periods within panels are indicated by different letters (below the lower left corner of each box; Tukey HSD, *p* < 0.05). Diamonds represent the mean. Sample sizes (number of months) are shown above the mean. UD areas, and mean fractions of optimal habitat are provided in Table 4

Significantly different period means (within seasons) are denoted by different superscripted letters (Tukey HSD, *p* < 0.05). Also see Figure 4.

The decrease in the average percent of monthly optimal habitat between periods was even greater within the 95% UDs (Figure 3). Optimal habitat within the 95% UD during the melt season declined across periods (*F*_2,60_ = 30.77, *p* < 0.001; Table 4), with all periods significantly different from the others (1985–1995:25.5%; 1996–2006:11.9%; 2007–2017:5.2%; Figure 4a, Table 4). A similar pattern occurred in the ice minimum season (*F*_2,39_ = 32.45, *p* < 0.001) as all periods were significantly different from the others (1985–1995:22.5%; 1996–2006:13.7%; 2007–2017:8.0%; Figure 4b, Table 4), and in the ice growth season (*F*_2,42_ = 51.59, *p* < 0.001), with, again, all periods were significantly different from the others (1985–1995:27.1%; 1996–2006:18.9%; 2007–2017:13.6%; Figure 4c, Table 4). Differences in the percent of optimal habitat across periods occurred in the ice maximum season (*F*_2,231_ = 37.01, *p* < 0.001), however, this was a result of a greater percentage of optimal habitat in 1985–1995 (35.1%) than in 1996–2006 (27.8%) and in 2007–2016 (25.0%; Figure 4d, Table 4). Only in the maximum ice season did two periods (i.e., 1996–2006 and 2007–2016) have a similar percentage of optimal habitat within the 95% UD (Figure 4d). Whereas declines in optimal habitat were most pronounced in the continental shelf regions of the Beaufort and Chukchi seas during the ice melt, minimum, and growth seasons, the decline in average percent of monthly optimal habitat during the ice maximum season was expressed largely in the Chukchi Sea (Figure 3).

**Figure 5:**
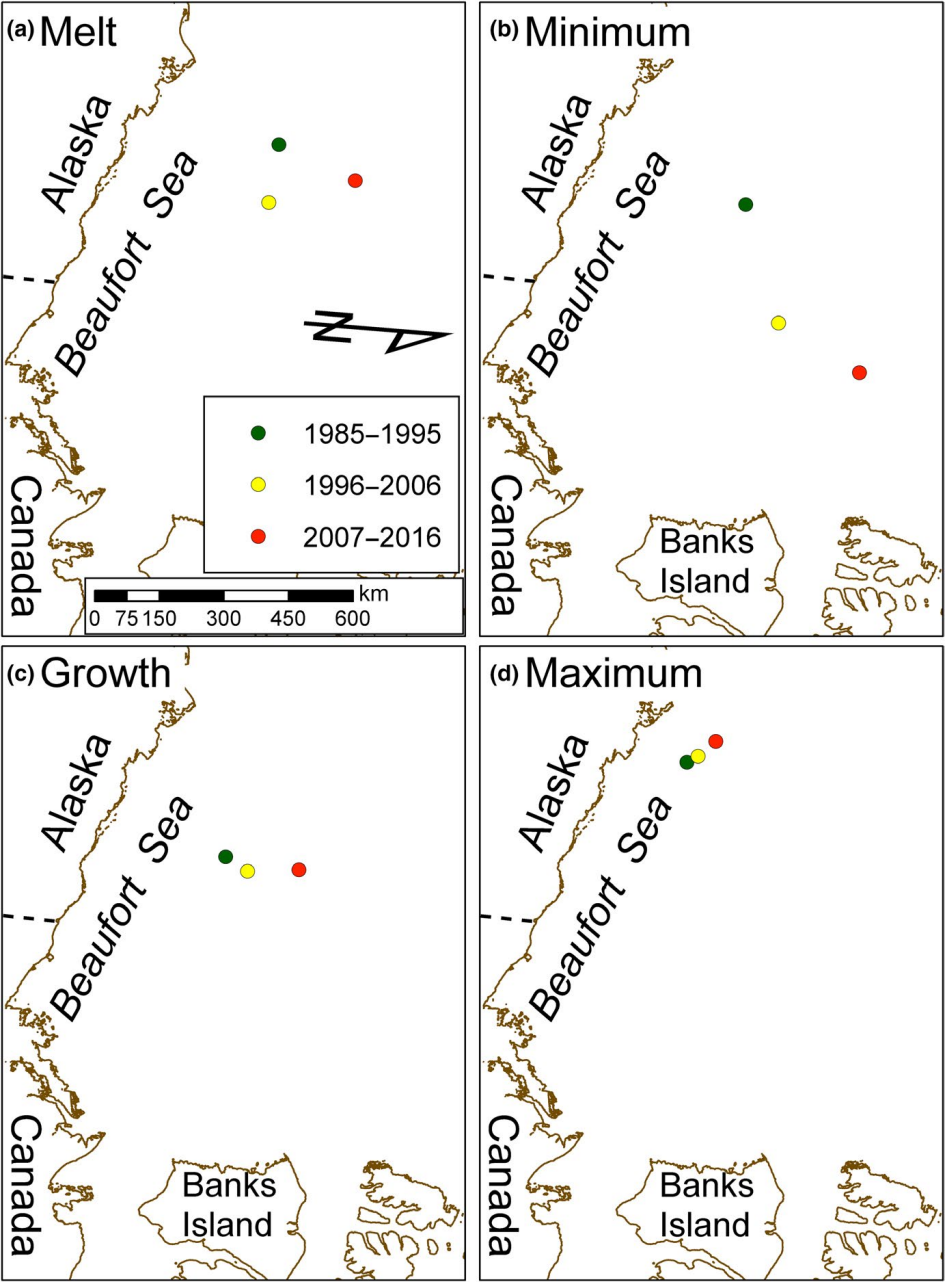
Polar bear optimal sea ice habitat centroids across 3 decades. Centroids for ice melt, minimum, and growth seasons generally moved north and east across decades. Centroids remained relatively unchanged during the ice maximum season across decades. Data values for this figure are provided in Appendix 2

Across periods, optimal habitat centroids generally moved northward and eastward in the ice transitional and minimum seasons but were relatively unchanged during the ice maximum season (Figure 5). The greatest change in centroid position was in the ice minimum season (Figure 5b). The minimum season centroid in 1996–2006 was 288 km (at a bearing of 61°) from where it occurred in 1985–1995, and in 2007–2016 was 223 km (at a bearing of 27°) from where it occurred in 1996–2006. In contrast, the movement of optimal habitat centroids during the ice maximum season was relatively small, and all remained near the Alaska coast (Figure 5d). The ice maximum centroid in 1996–2006 was 29 km (313° bearing) from its position in 1985–1995, and in 2007–2016 was 54 km (302° bearing) from its location in 1996–2006. Additional results on centroids are provided in Appendix 2.

## 4 DISCUSSION

Our assessment of Arctic‐wide seasonal RSFs from 1985–1995 when applied to environmental data in the Beaufort and Chukchi seas between 1996 and 2016 indicates that the efficacy of the reference RSF models to predict adult female polar distribution has diminished. In 1996–2006 and 2007–2016, and during seasons of ice melt, ice minimum, and ice growth, the distribution of polar bears and the distribution of optimal habitat showed relatively little overlap. During those seasons, as optimal habitat decreased and shifted north, polar bears expanded their range and used more sub‐optimal habitat. Utilization of suboptimal habitats can be maladaptive for wildlife (Hollander, Dyck, Martin, & Titeux, 2017). For polar bears, individual fitness could be negatively impacted as greater movements in search of suitable habitat increase energetic costs, and increased use of sub‐optimal habitats reduce success in capturing prey. For polar bear conservation, the reference RSFs developed by Durner et al. (2009) for the melt, minimum, and growth ice seasons appear less effective for predicting the distribution of polar bears under contemporary sea ice conditions in the Beaufort Sea region, but they are effective in illustrating some of the habitat challenges that polar bears face in a changing Arctic. During the ice maximum season in both 1996–2006 and 2007–2016, the 50% kernel UD was highly concordant with the distribution of optimal habitat in the Beaufort Sea and the distribution of that habitat remained relatively constant across the decades. Hence, the winter‐time RSF developed from data collected > 20 years ago still models polar bear distribution and optimal habitat accurately in the Beaufort Sea, confirming that the reference RSF for the ice maximum season continues to provide an effective tool to predict polar bear distributions and to inform conservation efforts.

Across all seasons and Arctic‐wide, Durner et al. (2009) reported that for the 1996–2006 decade, 97.5% and 82.3% of polar bear locations, respectively, occurred within the upper 50% and 20% of RSF‐valued habitat, suggesting robustness of the 1985–1995 polar bear RSFs to changes in sea ice conditions the following decade. Similarly, polar bear habitat selection in the Chukchi Sea also remained unchanged between periods that experienced sea ice loss (Wilson, Regehr, Rode, & St. Martin, 2016). However, since the ice maximum season dominates the annual cycle, it would have been a primary driver of the annual performance patterns in Durner et al. (2009), tending to elevate them and impart similarity across decades.  Our examination here of season‐specific patterns in polar bear occupancy of RSF‐valued habitat, and within a subregion of that examined by Durner et al. (2009), is informative as it exposes the ramifications of applying reference RSFs to decadal periods with different, as well as similar, environmental conditions.  Our results suggest that during the seasons of sea ice melt, freeze, or minimum, the reference RSFs performed poorly compared to the annual and Arctic‐wide performance reported by Durner et al. (2009). This was apparent even for 1985–1995, the period of model development, as the percentage of polar bear locations in the top 20% of RSF‐valued habitat within our study area during ice melt, minimum, and growth ranged from 37.4% to 49.1%, compared to the Arctic‐wide annual performance of 71.6% for the same period (Durner et al., 2009).  Additionally, the performance of the RSF declined across periods in the sea ice melt, minimum, and growth seasons.  But during the ice maximum season, the availability of optimal habitat has remained largely unchanged across the years and continues to comprise a large proportion of the area typically used by polar bears.

During spring ice melt, summer ice minimum, and autumn ice growth seasons, there was a diminishing amount of optimal habitat in our study area and within the kernel UDs of polar bear distribution (Figure 4). Also, what optimal habitat remained during the latter two decades became further displaced from the areas normally used by SB polar bears. Regions in our study area that consistently had optimal habitat during each period represented a small and decreasing proportion of the area typically used by polar bears after 1985–1995 (Figure 3). Optimal habitat in the southern Beaufort Sea during the recent decade (2007–2016) has all but disappeared in all seasons except the ice maximum (Figure 3). Optimal habitat has persisted more reliably in the ranges of the Northern Beaufort Sea and Chukchi Sea subpopulations of polar bears. We suggest that the dearth of optimal habitat during the non‐winter seasons is at least partly responsible for the increasing range of Beaufort Sea polar bears; there is simply little optimal habitat for bears to find and occupy. Most of the non‐winter sea ice habitat is now sub‐optimal, so any sea ice, regardless of composition or location that can provide a stable substrate to walk on may be a likely candidate for polar bear occupancy for lack of a better choice and that choice may be maladaptive (Hollander et al., 2017).

A changing Arctic has diminished the abundance of optimal habitat for SB polar bears during the ice melt, minimum, and growth seasons. The loss of optimal habitat in these seasons, coupled with increasing duration (Stern & Laidre, 2016), is likely a contributing factor to the observed declines in survival and abundance of SB polar bears (Bromaghin et al., 2015). However, we found that when sea ice attained its average winter extent, optimal habitat was consistently present during all 32 years of our study. We also found that the distribution of polar bears during the ice maximum season reflected the distribution of optimal habitat across all 32 years of study, conveying predictability in how bears select optimal habitat when it is persistently available. This predictability also suggests that efforts to estimate the abundance and trend of SB polar bears may be most effective during the ice maximum season, as optimal habitat and bear distribution coincides with the distribution of research efforts for this subpopulation (Bromaghin et al., 2015).

Our findings elevate concerns for the future status of SB polar bears as the transitional seasons of sea ice lengthen and the extent of optimal polar bear habitat during those seasons declines. Indeed, the first conservation criterion of the U.S. Marine Mammal Protection Act is that the health and stability of marine ecosystems be maintained at a level such that polar bears may persist as “significant functioning elements” within those systems (USFWS, 2016). Consequently, actions directed toward slowing global warming and preserving the duration of the ice maximum season will also preserve optimal habitat, and thereby benefit polar bears.

## CONFLICT OF INTEREST

The authors claim that they have no conflict of interest.

## AUTHORS' CONTRIBUTIONS

G.M.D. and D.C.D. conceptualized the study and developed the problem formulation. G.M.D. conducted all analyses. G.M.D., D.C.D., and T.C.A. reviewed and interpreted the results of all analyses. G.M.D. led the writing of the manuscript. All authors contributed critically to the drafts and gave final approval for publication.

## Data Availability

The data used in this research are available through the U.S. Geological Survey: https://doi.org/10.5066/P9ZRJ3XU.

## APPENDIX 1

Sample sizes (records/individuals), before and after removing locations <72 hr apart, of satellite telemetry locations from adult female polar bears captured in the southern Beaufort Sea and used to assess baseline period (1985–1995) resource selection functions

**Table:**

Season	1985–1995	1996–2006	2007–2016
Before temporal filter			
Melt	3,286/75	10,675/85	31,623/79
Minimum	2,308/70	3,312/64	11,037/51
Growth	2,255/72	6,945/78	14,923/69
Maximum	19,696/93	30,009/100	127,817/136
Following removal of locations < 72 hr apart			
Melt	930/75	1,790/85	1,106/78
Minimum	662/70	511/64	484/50
Growth	565/72	932/78	688/66
Maximum	4,054/93	4,408/97	5,488/127

## APPENDIX 2

Seasonal centroids (latitude/longitude) of optimal polar bear habitat in the southern Beaufort Sea during 3 decadal periods, and the distance and direction between centroids from one period to the next.

**Table:**

Period	Latitude (N)	Longitude (W)	Bearing to next (°)	Distance to next (km)
Melt				
1985–1995	73.624	152.990	82.0	137.1
1996–2006	73.752	148.627	32.1	209.0
2007–2016	75.385	152.114		
Minimum				
1985–1995	73.745	148.468	61.2	287.7
1996–2006	74.824	139.784	26.8	222.9
2007–2016	76.582	135.895		
Growth				
1985–1995	72.917	147.179	21.45	61.3
1996–2006	73.428	146.473	346.8	120.3
2007–2016	74.478	147.392		
Maximum				
1985–1995	72.062	153.223	313.3	29.5
1996–2006	72.243	153.857	301.5	54.2
2007–2016	72.493	155.237		
